# Black and minority ethnic groups and forensic mental health

**DOI:** 10.1192/bjo.2021.357

**Published:** 2021-06-18

**Authors:** Donna Arya, Charlotte Connolly, Beth Yeoman

**Affiliations:** 1 Thornford Park Hospital; 2 Thornford Park Hospital, Southern Health NHS Foundation Trust

## Abstract

**Aims:**

To review the existent literature base regarding Black and Minority Ethnic (BAME) groups care pathway into and experience of care and treatment within secure services. This includes any differences (between BAME and majority ethnic groups) in rates of sentencing, sectioning, length of stay, received treatment and use of restrictive practice. Our overarching aim is to highlight the severe lack of research in this area and the corresponding need for increased research to both consolidate and progress the existing evidence base in order to inform and improve culturally competent service provision.

**Background:**

Research suggests that BAME groups have an increased risk of involuntary psychiatric care, longer-stays within services and higher rates of re-admission. Several explanations have been proposed for this observed disparity, however few of these proposed explanations have provided sufficient or consistent supporting evidence.

**Method:**

A review of both quantitative and qualitative research regarding BAME groups within secure services was conducted. Approximately twenty journal articles, literature reviews and meta-analysis published between 1988 and 2019 were included. The current study should be considered a snapshot and not reflective of the full extent of published literature on the subject. For inclusion, studies should have been conducted in either a forensic mental health setting or a prison and differentiate a minimum of two ethnic groups

**Conclusion:**

Research suggest that BAME individuals continue to experience an increased risk of involuntary psychiatric care, longer stays within secure services and higher rates of re-admission. Whilst many explanations for this disparity have been proposed, few have provided adequate supporting evidence. The ongoing lack of research within this field has led to a limited evidence base from which to inform culturally competent practice. The research which has been conducted has tended to produce inconsistent findings, in part due to the reliance on small scale studies with limited generalisability. Research within this area has been further complicated by varying definitions of culture and ethnicity across studies, leading to some suggestion that the issue of ethnic inequalities and pathways to care, has been misconceptualised. This highlights a critical need for increased research efforts to:

Understand why BAME individuals are at increased risk of involuntary psychiatric care, and how this disproportionate risk can be addressed

Explore potential disparities in the care and treatment of BAME individuals within services and how this might impact upon higher rates of re-admission

Ascertain how best to improve culturally competent service provision.

